# Concretization as a Mechanism of Change in Psychodrama: Procedures and Benefits

**DOI:** 10.3389/fpsyg.2021.633069

**Published:** 2021-02-23

**Authors:** Aviv Kushnir, Hod Orkibi

**Affiliations:** Faculty of Social Welfare and Health Sciences, University of Haifa, Mount Carmel, Israel

**Keywords:** psychodrama, mechanism of change, change factors, psychotherapy, concretization, therapeutic factors

## Abstract

Concretization is a concept that has different meanings in different psychological theories and varying manifestations in different psychotherapies. In psychodrama, much of the available information on concretization draws on J. L. Moreno’s initial conceptualization, descriptive case studies, and interpretations in the various approaches. However, concretization has not been empirically studied as a concept or as a therapeutic mechanism of change. Therefore, the purpose of this qualitative study was to generate an empirically based conceptualization and operationalization of concretization as well as to identify its clinical benefits in psychodrama. To this end, semistructured in-depth interviews were conducted with seven experienced psychodrama therapists. Using a grounded theory approach for the data analysis, the model that emerged consists of three pathways toward concretization: realistic concretization, symbolic concretization, and integrated concretization. The findings suggest a sequential multistep operation that can be linear or nonlinear, depending on the protagonist’s need. The findings also underscore four benefits of concretization as a mechanism of change in psychodrama: reducing the ambiguity of the problem, externalizing the protagonist’s problem, enhancing the therapist-protagonist therapeutic bond, and bypassing the protagonist’s defense mechanisms. The model is discussed in light of the findings and the literature, and future directions are suggested.

## Introduction

Psychodrama is an experiential psychotherapy in which guided role-play is used to gain insights and work on personal and interpersonal problems and possible solutions (Orkibi and Feniger-Schaal, 2019). One of the key core mechanisms of change in psychodrama is concretization, which so far has not been empirically studied. Therefore, the purpose of this qualitative study was to generate an empirically based conceptualization and operationalization of concretization as well as to identify its clinical benefits. The term concretization comes from the verb to “concretize” that means “to make something concrete, specific, or definite” (Concretize, 2020). Concretization has appeared in various psychological theories over the years. Some scholars have used the term concretization explicitly, though without necessarily providing a definition, while others have alluded to its meaning without explicitly using the term. The next section presents several examples of the diverse uses of the term and the resulting variations of meaning.

### Concretization in Psychological Theories

To the best of our knowledge, while Sigmund Freud did not use the term concretization explicitly, the fundamental meaning of the term is implied in Freud’s psychoanalytic theory. First, Freud’s theory of the human psyche (i.e., personality) is conceptualized as having a tripartite structural system consisting of the superego, the ego, and the id. This structural system can be seen as another example of Freud’s use of concretization to differentiate between parts of the client’s intangible psyche (Freud, 2018). Second, in practice, when a client’s suppressed emotions are unconsciously displaced onto the therapist (or vice versa), transferential content takes on the concrete and specific form of the “here and now” in the therapeutic relationship (Freud, 2018).

Another example can be found in the work of Milton Erickson, who used metaphorical stories as indirect pathways to his clients’ psyche (Erickson and Havens, 1996). According to Erickson, access to clients’ repressed psyche material requires a vehicle that can bypass ordinary verbal communication. Metaphorical stories not only are rich in information and elements but also enable the client to express a mental event in a distant and reserved way that ordinary words cannot. Thus, a metaphorical story can be seen as a concretization of abstract material from the client’s psyche that can be identified and analyzed with the therapist (Erickson and Havens, 1996).

In contrast, Piaget (1974) explicitly used the term “Concrete Operations” to explain the third stage in his theory of cognitive development. This stage takes place between the ages of 7 and 11 years and is characterized by the development of organized and rational thinking. In this stage, children can only apply logic to physical objects (hence “concrete operational”), and they are typically not yet able to think abstractly or hypothetically. Thus, Piaget used the term “concrete” to describe a child’s basic ability to analyze a tangible reality that is physically given. Along these lines, concretization in psychodrama may possibly enable the client to gain action insights into an abstract inner experience.

### Concretization in Psychotherapies

The concept of concretization has also been integrated into certain psychotherapy methods and has acquired various meanings and forms. The following section presents examples of concretization in other forms of psychotherapy.

Emotion-focused therapy (EFT) is an empirically supported treatment approach that focuses on deep emotional processing, including increased emotional awareness and regulation, as well as the transformation of maladaptive emotional responses to personal and interpersonal issues, such as the blockage of emotional experience and expression (Greenberg and Watson, 2006). Two central procedures that have been used in the EFT facilitate concretization. One is the “empty chair dialogue” for unfinished business and negative feelings toward a significant other. The other is the “two-chair dialogue” when one aspect of the self is in opposition to another aspect (Greenberg, 2015). Note that the use of a chair in therapy was borrowed from psychodrama and Gestalt (Moreno and Fox, 1987, p. 131).

Cognitive behavioral therapy (CBT) is a structured, short-term, present-oriented psychotherapy directed toward solving current problems and modifying dysfunctional thinking that produces an improvement in mood and behavior (Beck, 2011, p. 2). CBT includes several techniques for self-monitoring to transform inner automatic thoughts and feelings into overt and external ones (at the core of behavior patterns) and to differentiate the person from their feelings and thoughts. In this sense, the separation and the flexibility of the relationship between clients and their thoughts and feelings are made possible by the process of concretization using the tangible written documentation of inner cognitions and emotions (Beck, 2011).

Narrative therapy is based on separating clients from their problems and thereby replacing a dominant problem-saturated narrative with an alternative narrative (White and Epston, 1990). Allowing clients to externalize their problems rather than internalize them gives the client a new perspective on the problem while identifying new positive facts that have been engulfed by the overall negative experience. Externalization of problems enables clients to create a multidimensional dialogue on their issues and re-author their narrative (White and Epston, 1990).

In the creative arts therapies, Blatner (1991) suggested that *to concretize* means “to change an abstract statement into something more concrete, which can be perceived by looking at a particular situation or by a physical experience of the emotion associated with that situation” (p. 406). Creative arts therapies can gradually bring up abstract themes and present them more tangibly in various ways, such as through the use of colors, shapes, and composition in Art Therapy, the embodied use of dramatic action in Psychodrama/Drama Therapy, the use of physical movement, posture, and gesture in Dance Movement Therapy, the use of sound, voice, and playing instruments in Music Therapy, and the use of written expression in Bibliotherapy. This may enable clients to experience their subconscious dynamics more colorfully and concretely while providing access to pre-verbal experiences. Clients’ external observation of their internal experience may increase their ability to reflect on and analyze themselves as well as receive feedback from the therapist and members in group therapy (Blatner, 1996).

Overall, as in psychological theories, different approaches to treatment also apply different definitions of the term concretization. One of the commonalities across these methods is the need for specificity to create a concrete separation – physically or mentally – between the client and his or her problem.

### Concretization in Psychodrama

The following section describes various conceptualizations and applications of concretization in the psychodrama literature. Jacob Levy Moreno, the creator of psychodrama, defined and explained concretization in psychodrama theory and practice. At the theoretical level, Moreno viewed group therapy and sociodrama as methods that make it possible to concretize daily situations and social phenomena from a human experience (Moreno and Fox, 1987, pp. 7, 18–19). On a practical level, Moreno addressed concretization in the different phases of treatment through the creation of the “here and now.” According to Moreno, this dimension is part of the “surplus reality,” i.e., a dramatic reality that is the subjective extension of the client’s everyday reality, where the client’s inner feelings and thoughts can become present, visible, and tangible. Moreno suggested that “one of the basic instruments in constructing a client’s psychodramatic world is that of the auxiliary ego, which is the representation of absentee individuals, delusions, hallucinations, symbols, ideals, animals, and objects. They make the protagonist’s world real, *concrete*, and tangible” (p. 9; emphasis added). In addition, Moreno referred to a range of ways to help clients explore intrapersonal and interpersonal issues and make them concrete, tangible, and external, including role-playing, auxiliary ego, and the drawing of a social atom map (Moreno and Fox, 1987).

Therefore, concretization lies at the heart of psychodrama. In fact, on the psychodrama stage, the therapist offers the clients ways to represent their overt and covert life (Farmer, 1995). The therapist, together with the client, can give physical expression to real things from the client’s life, such as objects, characters, places, and times. Similarly, a physical expression can be given to abstract things from the client’s life, such as feelings and thoughts, for example, by concretizing the experience of feeling trapped, burdened, suffocated, or oppressed by something. As highlighted by Farmer (1995), “Through the director’s exploitation of metaphor, anything can represent anything else, and by a process termed “concretization,” ideas or images may be put into spatial dimensions in the form of objects or people on the stage” (pp. 14–15).

Holmes (2015) explained that through the lens of attachment theory (Bowlby, 1988), internalized object relationships can be vividly externalized in two main ways. One is in the client-therapist transferential relationship and the other is on the psychodrama stage when childhood scenes are enacted. Such concretizations of the original dyadic relationship can help explore their effects on the client’s adult relationships in the here and now (Holmes, 2015, p. xviii).

Blatner (2000) defined concretization in psychodrama as a work that helps the protagonists to convert their abstract statements and metaphors into specific life scenes (Blatner, 2000, p. 238). Blatner also points out two main forms of concretization. One involves transforming a general issue that the protagonist experiences (e.g., “conflict with authorities”) into specific scenes on the stage of psychodrama (e.g., a conflict with the boss). The second involves converting metaphors that arise in therapy (e.g., “I feel trapped”) into a tangible enacted reality on the stage (e.g., auxiliary egos enact a barrier around the protagonist; pp. 238–239).

According to Vander May (1981), who mainly wrote about individual psychodrama, “concretization is the process of making visible those invisible and often elusive qualities of the dynamic interflow that occurs between an individual and other people, animals, objects, and one’s self. It brings to life ideas, feelings, and concepts by giving them substance” (p. 35). Vander May explains that concretization is often useful in situations where the client tends to intellectualize and where the therapist’s role is divided into two stages. First, the therapist must listen attentively to the client’s words, identify and translate them (the verbal and nonverbal information) into an image. In the process of choosing the path to concretization, the therapist must consider many factors, such as height, size, location, sound, color, light, texture, temperature, body language, and symbolic language. Then, the therapist can move to the second stage using a role-playing technique and inviting the client’s image to become real on stage.

Fonseca (2004) introduced “the concretion technique of sensations and feelings …. [that] happens through body postures or through the placement and pressure of the client’s hands over regions of his own body” (p. 79). Concretization is central to psychodrama because it facilitates the client’s identification of various facets of personality. The self-observation of the client on frequently repressed facets is the result of a joint effort by the therapist and the client to concretize the same internal material. This joint effort includes analysis of the client’s body language and verbal messages that together reveal conscious and unconscious material (Fonseca, 2004).

According to Kellermann (1996), “‘concretization’ may play a part both in the onset, and in the removal of somatoform symptoms” (p. 149).^1^ Kellermann emphasizes the complexity of using the concretization technique in treating clients with somatic disorders and the damage that can be done in case of misidentification of the psychosomatic process in the psychodrama treatment and miscomprehension of the influence between the client’s mind and body. The therapist must be aware that incorrect guidance using concretization of inner blocked parts of the client’s psyche may worsen the client’s physical symptoms (Kellermann, 1996). The dialogue that the client maintains with these inner blocked parts must be pursued with caution, the required moderation, and by providing a sense of control to the client. One of the main purposes of Kellermann’s remark is to assist the therapist when dealing with uncertainty with respect to the content that may arise during clients’ concretization, the choice of their representation, and the manner in which clients cope with them. The use of concretization in psychodrama makes it possible to expand clients’ control and understanding of these inner parts of their psyche, without increasing their sense of powerlessness as regard these inner parts (Kellermann, 1996).

In summary, the different psychodrama approaches to concretization reflect differences in applications and definitions of the term. Some view concretization as a technique and others as a dynamic therapeutic process. In most approaches, concretization is an inseparable part of psychodrama theory and practice, which enables the client to access internal materials (some abstract and unconscious) and to transform them into external parts that have a real form on the therapeutic stage. Through the therapist’s use of concretization, clients can observe and expand their perceptions of personal and interpersonal issues. Given the relatively broad interpretation of the term concretization, it is difficult to suggest a single (operational or conceptual) definition of the term, and to the best of our knowledge, there is paucity of case studies and clinical studies that have focused on the effects of concretization in the therapeutic process and their expression.

### Purpose of This Study

Given the lack of empirical studies on concretization in psychodrama, the overarching purpose of this qualitative study was to generate an empirically based conceptualization and operationalization of concretization. The findings can provide valuable information not only for clinicians but also for future experimental studies where concretization can be operationalized and its impact as a specific therapeutic mechanism can be tested. This study specifically aimed to (1) generate a conceptualization of concretization in psychodrama, (2) generate an operational definition for future experimental studies, and (3) contribute to a better understanding of the therapeutic effects of concretization as perceived by seasoned psychodrama therapists. Accordingly, the research questions were: (1) how is concretization conceptualized and implemented by seasoned psychodramatists in their practice? (2) how do they perceive the therapeutic value of concretization? and (3) in what ways is it possible to operationalize concretization for future experiments?

## Materials and Methods

This qualitative study implemented the constructivist grounded theory method (Charmaz, 2015), which was chosen given the lack of empirical studies on concretization in the psychodrama literature, for “grounded theory is well known as a method that can be employed where existing theories or areas of research are under-defined or patchy” (Tweed and Charmaz, 2012, p. 134). In addition, researchers recommend that qualitative definitional and descriptive research in psychotherapy should precede quantitative outcome research reasoning: “it is always good to know what something is before you try to measure how much of it is present” (Rodgers and Eliott, 2015, p. 560).

### Procedure and Participants

After approval by the Ethics Committee for Human Research at the University of Haifa (approval # 19/139), a list of potential interviewees was created. The list included contact information of seven psychodrama therapists with more than 10 years of experience in training and supervision who were contacted by the first author to participate in the study anonymously and voluntarily. All seven psychodrama therapists agreed to participate and signed an informed consent form where it was specified that the interviews would be recorded and transcribed with no identifiers. See Table 1 for the participants’ background information.

**Table 1:** Participants’ Background Information.

Participant	1	2	3	4	5	6	7
Gender	F	F	F	M	F	F	F
Residence in Israel	Center	Sharon	Center	Sharon	North	Center	North
Psychodrama training	Moreno’s institute in Beacon, NY. MA and PhD degrees	MA degree	MA degree	MA degree	Certificate (MA in creative education)	MA degree	MA and PhD degrees
Years of practicing psychodrama	Over 40 years	Nearly 30 years	Over 25 years	19 years	24 years	22 years	Nearly 20 years
Main work	Psychiatric hospital, special education, private groups, and some individual, teaching, supervision	Addiction treatment unit, individual treatments, teaching, supervision	Psychiatric hospital, therapy center, private clinic, teaching, supervision	Prisons, hospitals, private clinic, teaching, supervision	Schools, private clinic, teaching, supervision	Psychiatric hospital, private clinic, teaching, supervision	Psychiatric hospitals, general hospital, private clinic, teaching, supervision
Client populations	People with mental illness	Children, students, addicts and their families, elderly	People with head injuries, mental illness, hospital staff, teachers	Prisoners, youth, bereaved families, clients with muscular degeneration and cancer, ultra-orthodox Jews	School children, adults	Mental health (varying diagnoses), youth, women, and men	Youth at risk, people with psychiatric diagnoses, autism spectrum
Experience as trainer/educator	40 years+	10 years	25 years	15 years	6 years	20 years	8 years

### Interviews

A semistructured individual interview was conducted with each psychodrama therapist. An interview guide with open-ended questions was used. The interview questions were designed to cover a wide range of experiences but at the same time be narrow enough to elicit and explore the participants’ specific views and experiences. When conducting the interviews, an effort was made to understand the participants and the concepts they used to formulate their perceptions of the topic (Charmaz and Belgrave, 2012). The interview included background questions (e.g., What is your main place of employment as a psychodramatist?) and questions about concretization d (e.g., In what situations do you apply concretization and how?).

### Data Analysis

Data analysis followed Charmaz (2015) grounded theory approach, which is underpinned by a constructivist paradigm that is ontologically relativist and epistemologically subjectivist. This approach acknowledges multiple perspectives and the co-construction of experience and meaning by the researcher and participant. The first stage of data analysis involved subjecting the interviews to line-by-line *initial coding* of gerunds to define the actions or events described by the interviewees. This was followed by the second phase of *focused coding* of “the most significant and/or frequent earlier codes to sift through large amounts of data” (p. 70). This phase of coding is more focused, fine-grained, and conceptual than the previous one. Through focused coding, we built and clarified each category by exploring all the data it covered and by identifying variations within it and between other categories. The data analysis processes included *theoretical sampling*, which is one of the tools that allows the researcher to examine and develop the categories that were created by searching for new data by re-questioning the participants or new participants, as needed. This simultaneous data collection and analysis is a major part of developing analytic codes and conceptual categories from the data while using *constant comparisons* of data with data, data with codes, codes with codes, and codes with categories to find similarities and differences.

Throughout the entire study, *memos* were written by the first author to examine, compare, and analyze the data, codes, and emergent categories. Written memos were an important tool to develop ideas and theories in the early analytic process. In the final part of the analysis, we constructed a model of concretization to better understand and account for the concept and process as well as its possible operationalization. The various parts of the research process, including the relationships between the categories, were visualized in a diagram (Charmaz, 2015). A member-checking procedure (i.e., participant validation) was conducted with two interviewees to assess the credibility of the findings, which resulted in slight clarifications (Creswell and Miller, 2000).

## Results

### The Two Types of Concretization

#### Realistic Concretization

Concretization as a mechanism of change in the realistic dimension facilitates the transition of the selected content into external and realistic representations through a dramatization of self-presentation from the protagonist’s recent, past, and anticipated future life events. This is reflected in the therapists’ descriptions: “There is the realistic concretization that produces the framework for the therapy… and afterwards, there is a concretization of the protagonist’s inner parts” (Therapist 7). Another therapist explained:

Many times, concretization begins with a realistic scene. This scene, as much as we may try to refine it and re-create it, will not be [presented] exactly as it occurred in real life. Something new happens and then there is a chance for different content to emerge from the protagonist (Therapist 6).

A different therapist stated:

During this process of the protagonist’s physical work of constructing the scene and making the stage realistic, he [the protagonist] undergoes a process of warming up. The purpose of concretization at its basic level is to elicit the protagonist’s spontaneity and bring him into the here and now … the therapist looks at how the protagonist builds the realistic scene: what are his priorities?… You get a lot of important information during the concretization process of a place, which may appear to be simplistic (Therapist 2).

In sum, concretization in the realistic dimension focuses on the reconstruction and dramatization of a realistic scene as a step toward proceeding to the core of the protagonist’s problem.

#### Symbolic Concretization

Concretization as a mechanism of change in the symbolic dimension allows for the transformation of the protagonist’s abstract or suppressed inner content into a tangible and external representation. This transformation can be achieved by gradually proceeding from the periphery to the center of the protagonist’s problem. The transformation of content can potentially take place at the beginning of a therapy session if the protagonist is warmed up to work and has high awareness of the content. This can be manifested in the protagonist’s ability to describe the content emotionally through an image at the beginning of the session. This is illustrated in one therapist’s explanations:

Sometimes, concretization begins with a realistic scene that produces a realistic image, or non-realistic concretization may produce an unrealistic image such as when a family that puts an emotional burden on the protagonist. In the group [therapy], we would create a representation of this burden, with the help of other participants, which will physically put the burden on the protagonist and weight him down (Therapist 6).

A different therapist commented:

Concretization contributes to symbolic work because many times the concretization is not just about creating the scene. Sometimes, I [the therapist] can say [to the protagonist]: “you are always talking about this bad relationship and the anger that comes out of you, let us give an image to the anger.” Then you create it [the image of anger] with the protagonist. At first, we examine colors, softness, roughness, then we’ll give it a voice and explore how it affects the protagonist (Therapist 7).

Another therapist explained: “Metaphors are a concretization of the abstract. You take very abstract content, diffused material … and concretize it through metaphor… working through images makes the therapy more accurate” (Therapist 3).

In sum, concretization in the symbolic dimension focuses on the transformation of the protagonist’s inner and sometimes repressed content into a physical representation during therapy. These two types of concretization thus point to a variety of ways to produce a concrete representation, including the use of group members, objects, and the verbal expression of images, which are particularly useful in individual therapy.

### The Operation of Concretization as a Mechanism of Change

The interviewees pointed out that concretization as a mechanism of change may include four operations that can stand alone as a separate therapeutic intervention that can be implemented according to the protagonist’s needs. The four operations consist of capturing a potentially emotionally loaded (i.e., charged) content from the brief interview that follows the warm-up phase, the shared observation of the protagonist’s represented content, the transformation of abstract content into tangible, and the external representation of the protagonist’s insight.

#### Capturing Potentially Emotionally Loaded Content From the Brief Interview

In one operation of concretization as a mechanism of change, the therapist gathers as much information as possible about the protagonist’s problem during a brief interview that follows the warm-up phase and before moving to the action phase. The therapist pays close attention to content that is potentially emotionally loaded (i.e., charged) as conveyed both verbally and nonverbally such as the use of imagery, metaphors, physical experiences, analysis of body language and gestures, type of breathing, gaze, or repetition of words. Next, the therapist and protagonist decide jointly how to externally represent the internal content. Note that, in groups, the representation is done by the group members and, in individual therapy, the representation is usually done through objects and verbal expression of images. This is supported by a therapist’s examples:

Another thing that helps me to concretize is trying to capture metaphors or visual images when interviewing the protagonist. For example, the protagonist feels that he is constantly “on a cliff edge.” This is how the scene would begin – the protagonist stands on the cliff… This will often advance our [the therapist’s] ability to concretize in psychodrama (Therapist 3).

Another therapist echoed this idea:

Sometimes, the symbolic concretization will come straight after the interview. I can ask him about an image or picture that illustrates the feeling or situation he is trying to describe. And if it’s too hard for him [the protagonist], I [the therapist] can share the image that came to my mind to see whether it’s suitable (Therapist 6).

This idea was further clarified by another therapist who said:

As a psychodrama therapist, you must ask yourself: What do I hear? What do I see? And then how am I going to concretize this during therapy? I have a lot of options at this phase. For example, it would be natural to focus on the protagonist’s story and concretize it accordingly, but sometimes both the therapist and the protagonist can decide to concretize the protagonist’s fears [of the therapy process] and their physical expression during the interview (Therapist 4).

Similarly, another therapist explained: “Concretization is an actual experience that takes place in the actual present… It’s similar to the child’s experience [of imaginary play]: this chair is not just a chair, but anything I [the protagonist] want it to be” (Therapist 5). Further reinforcement for this idea can be seen in another therapist’s example: “I am having dinner with my family, everything goes well, and then I go into my room and I freeze.” And I [the therapist] ask him to show me this experience in the here and now (Therapist 7). In sum, this operation focuses on the therapist’s careful attentiveness to the protagonist at the beginning of the therapy session and his or her ability to identify verbal and nonverbal content that can become a concrete representation.

#### Shared Observation of the Protagonist’s Represented Content

In the second operation, both the therapist and the protagonist observe the representation of the protagonist’s content by using the “mirror” technique when the protagonist is played by a “double” and observes it from the audience. This “distanced” observation allows both the protagonist and the therapist to capture the current emotions that the protagonist is expressing when observing the scene from the outside that is being played. This process is illustrated in the following therapist’s examples:

I can freeze the protagonist’s scene and ask him: “What do you feel now?” [The protagonist answers:] “I feel that I am dazed, and I cannot find words because of my mom’s screams and that’s what’s happening to me… The therapist and protagonist observe the protagonist’s feeling and move from the current scene to the representation of that feeling (Therapist 7).”

This idea also can be seen in another therapist’s statement: “The protagonist creates a sculpture with some awareness, but observing this sculpture [from the outside] creates the action insight. After that, he [the protagonist] looks at this sculpture, and things that he was unaware of jump out. Emerge” (Therapist 5). Another therapist explained:

The most important thing in the initial phase of protagonist’s physical work or the building of the scene is my observation as a therapist of everything the protagonist is doing and that everything that happens is meaningful. My thoughts and insights as a therapist will be formulated after our joint observation of what is happening (Therapist 6).

This idea was also reinforced in another therapist’s explanation: “As therapists, we accompany the protagonist, learn his unique language, observe what is happening along with him, and at that moment become his ‘double’” (Therapist 1). In sum, this operation focuses on the therapist’s and protagonist’s observation of content as a way to capture emotions and proceed to the core of the protagonist’s problem.

#### The Transformation of Abstract Content Into Tangible

In another operation, the therapist and the protagonist create a tangible and physical representation in response to the emerging content. The focus is placed on what the protagonist needs and is capable of doing. This concept of transformation is illustrated in the next therapist’s explanation:

When you concretize abstract content, you deal with repressed and elusive content. The use of metaphor is one way of concretizing these contents. For example, ‘I have butterflies in my stomach.’ The transformation from repressed emotion into a concrete representation can occur through the use of a metaphor. This concrete representation can help the protagonist expand his understanding of the problem he brought to therapy (Therapist 3).

Another therapist mentioned this idea as follows:

Concretization turns the protagonist’s experience into something more accessible for understanding and coping. It’s no longer me [the protagonist] fantasizing inside my head that I’m nothing – it’s outside and it’s possible to represent this abstract experience of being nothing; it becomes concrete, tangible, and present (Therapist 5).

A different therapist explained: “We create an external representation according to what the protagonists tell us based on their physical behavior and taking into account my experience as a therapist dealing with this content” (Therapist 4). In sum, this operation focuses on the transformation of the protagonist’s abstract and hidden content into a tangible and physical representation. This transformation involves the ability to give shape to abstract content that needs to be explored.

#### Creating Representation of the Protagonist’s Insights

In the last operation, which is based on new insights from the protagonist’s psychodramatic exploration, both the therapist and the protagonist create a physical representation of this insight. In this context, the two create a realistic or abstract scene that enables the protagonist to internalize the insight through psychodramatic action. This is illustrated in one therapist’s explanation:

The protagonist internalizes this new experience he achieved in psychodrama, the insight does not remain theoretical without a substantial anchor, we experience it. Let us say he [the protagonist] does a role-play with his father and his father hugs him. The father hugs him, but the protagonist understands that he needs a physical hug and compassion from himself. With the help of the auxiliary ego, the protagonist experiences this insight physically and this helps him internalize it (Therapist 2).

This idea echoes the view of another therapist:

I remember a protagonist working on her relationship with her sister who had a cognitive disability… She realized the relationship had burdened her since childhood… The new insight gave rise to a dialogue between the two that was not possible until that therapy session… The “surplus reality” must come from the action we have taken, and the insights we have gained, and not from thoughts and words that were there before the psychodrama session (Therapist 3).

This notion is demonstrated in another therapist’s comment: “you can analyze or define lots of things, but there is a fundamental difference between understanding [intellectually] and gaining insights. Insight is comprehension based on experience, which helps to become aware of something new [about the issue]” (Therapist 4). In sum, this operation focuses on creating a concrete representation of the protagonist’s insights to help internalize them.

### The Benefits of Concretization

Several specific benefits emerged from the data as the result of using concretization as a mechanism of change.

#### Reducing Ambiguity by the Physical Representation of Content

Concretization is posited to be the external and physical representation of the protagonist’s problem. The physical appearance of the problem provides the protagonist with initial relief, since it makes it possible to capture the problem, observe, identify, name, and define it. It can be argued that concretization in psychodrama is akin to the photographic process. In both cases, a single moment or single content is captured, which contains a wider story that now becomes more accessible and observable. This emerged in one therapist’s explanation: “The mere act of externalizing the protagonist’s problem and the fact that it takes shape and life is part of the healing” (Therapist 5). This concept was also echoed in another therapist’s observation:

Even if we created a concrete form for the content and we did not work on it beyond that, I think it can provide some relief or calm the protagonist, and possibly in another psychodrama [session], the protagonist will be able to work beyond that (Therapist 2).

Additional therapist said: “We offer him [the protagonist] a new language that will help him express his problem, and simply expressing the content makes things easier and is part of the therapy process” (Therapist 1). A different therapist noted: “The very fact that you [the protagonist] observed the problem tangibly on stage, allowed new things being revealed that help in the therapeutic process” (Therapist3). In sum, this representation allows the protagonist a new perspective on the content and can provide immediate relief, even before further or deeper interventions have taken place.

#### Externalizing the Protagonist’s Problem

Concretization as a mechanism of change serves to separate the protagonist from the problem and lets him or her view it through a physical representation. This was illustrated in one therapist’s explanation: “Concretization allows the protagonist to view himself from a distance and in a different way. It is possible to get to new places that may shed light on the protagonist’s story and introduce new information” (Therapist 6). Similarly, this notion was described by another therapist: “I’m talking about the possibility of creating an external and real representation of emotions, conflicts in relationships or roles” (Therapist 7). Additional therapist explained: “It is finally possible to create a form for it [protagonist’s content] and to allow it to exist and not only in the protagonist’s mind” (Therapist 5). This concept can also be seen in another therapist’s words:

Many times, concretization makes it possible to externalize different parts of me [the protagonist], different parts of myself, and through role-playing, I [the protagonist] can observe the concrete content and ask myself: What do I feel now? What can I see now? (Therapist 3).

In sum, concretization as a therapeutic operation allows for the separation and externalization of the protagonist’s content. The protagonists can then be differentiated from their problem and observe it from the outside and experience it in a new way.

#### Enhancing the Therapist-Protagonist Therapeutic Bond

Concretization enables both the therapist and protagonist to stand together inside the protagonist’s dramatic scene. This shared position allows the therapist and protagonist to observe the externalization of the protagonist’s inner content and, in a way, to experience the dramatic occurrence together for the first time. The therapist is actively involved in externalizing the protagonist’s content, allowing the therapist to be physically close to the occurrence while observing the scene alongside the protagonist – often even from the same body pose. This gain of concretization likely contributes greatly to the formation of the therapeutic bond between the protagonist and therapist and helps the latter intervene accurately during therapy.

As one therapist explained:

We are both on the same stage and see the same things, and not that I am] the therapist] above and the protagonist is below… In this place, we can have a dialogue… The protagonist can accept me without seeing me as an intimidating, frightening, or judgmental authority. It’s easier for him to accept me (Therapist 2).

This notion was also mentioned by another therapist:

I [the therapist] will really be with him [the protagonist] in this place, my focus as a therapist will not be on how I break his [the protagonist] resistance or where I want to take the concretization. It may be okay to say that it is not appropriate to move forward at the moment and then we will decide to leave it and stay it at the point in the specific session (Therapist 4).

Another therapist’s observation:

I think that the more we concretize, the more we avoid relying on psychological theories, and instead, we ask concrete questions. This work method allows the protagonist to move closer to himself and at the same time bring us [the therapists] closer to him (Therapist 4).

In sum, concretization allows the therapist and protagonist to get closer and view the protagonist from a similar physical and emotional viewpoint. This closeness can enhance the protagonist’s ability to trust the therapist, enhance the therapist–protagonist therapeutic bond, and at the same time allow the therapist to see and experience the protagonist’s content in a clear and accessible way. This unique position may help the therapist direct a more accurate therapy process.

#### Bypass the Protagonist’s Defense Mechanisms

Sometimes, when protagonists reach the action phase of psychodrama, without being sufficiently warmed up, their defense mechanisms may emerge. Participants may have different defenses, with different intensities and different levels of awareness of them. Concretization as a mechanism of change may allow the protagonist to access repressed content and can make the therapy more open and easier. The protagonist’s exposure to new content can take place when the protagonist acts concretely and physically in psychodrama therapy. When the protagonist works through role-playing and is assumed to be moving away from the current position, the opposite may occur. This idea was addressed by a therapist as follows: “The ‘Magic if’ in drama or theater is when I’m busy embodying someone else in a concrete way, I release my defense mechanisms… and that’s when I’m most myself” (Therapist 4). Similarly, a different therapist said:

Concretization releases the protagonist from many restricting thoughts that are replaced by concrete thoughts. Instead of thinking about “How am I going to perform? What am I going to do?” I ask myself “What does the living room look like? Where is the phone located? Where do I stand in the living room?” These concrete actions free the protagonist to bring up content s/he did not intend to, and this is when the protagonist’s defense mechanisms are lowered (Therapist 7).

This idea can also be seen in another therapist’s explanation: “In concretization, you let go of thinking and move forward by trusting your ‘gut feeling’ from less familiar places; then it is possible to touch an emotional space, to touch the core of experience” (Therapist 3). Another therapist noted:

Through concretization, we [the therapists] sometimes help the protagonist pay attention to the projections he is making and to differentiate between the inner subjective story and the story in the objective reality. This allows the protagonist and us [the therapists] to be precise and see things in a cleaner and more defenseless way (Therapist 2).

In sum, concretization as a mechanism of change allows the protagonist to bypass restrictive defense mechanisms and reach the core of the problem. This can make the therapy more open and easier for both the protagonist and therapist.

## Discussion

**Figure 1:**
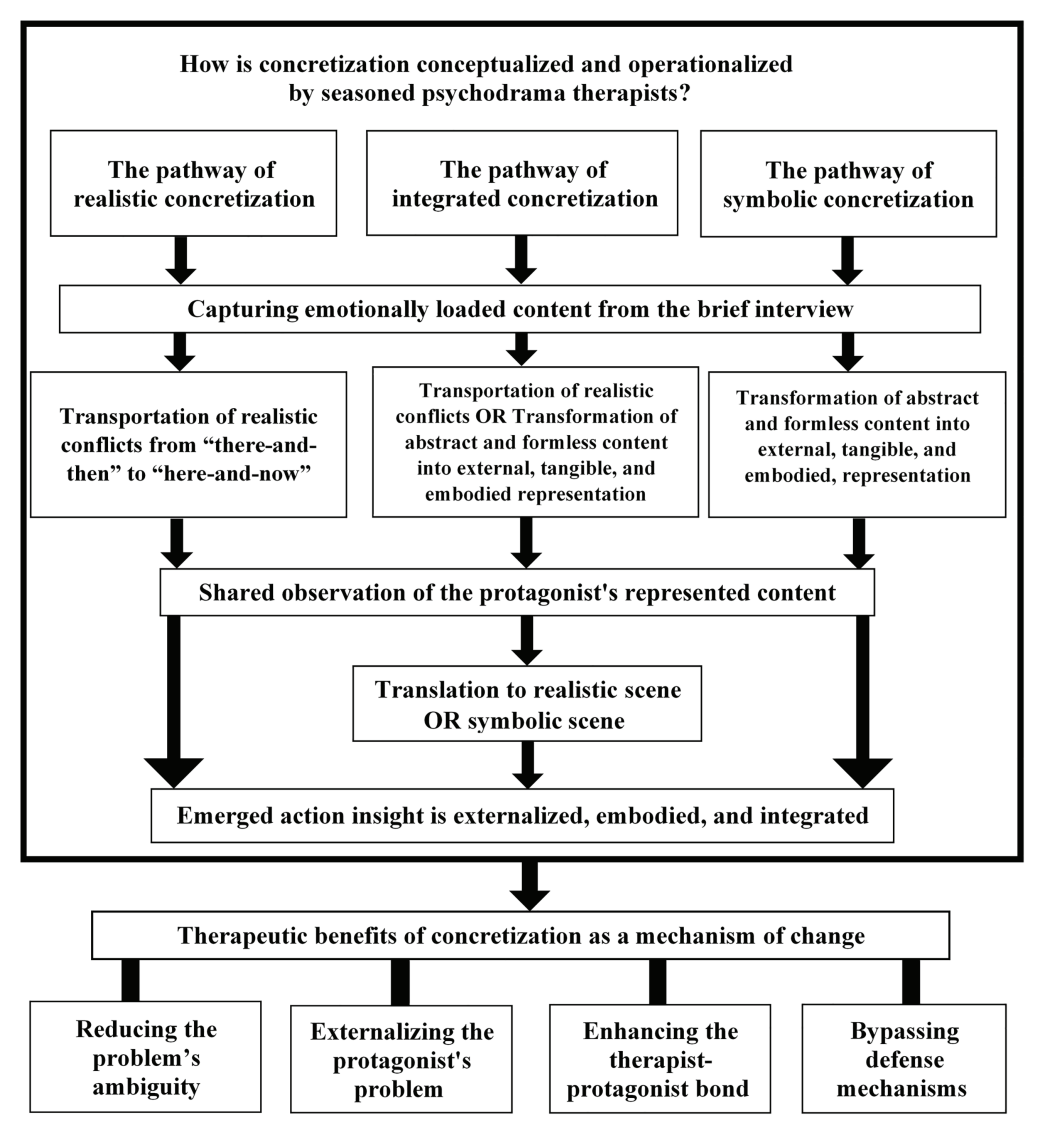
The Three Pathways of the Concretization Model.

The purpose of this study was to generate an empirically based conceptualization and operationalization of concretization for future experimental studies, as well as to better understand its clinical benefits as perceived by seasoned psychodrama therapists. In this section, we discuss the findings and the model that emerged from them as shown in Figure 1.

Our model consists of three possible pathways of concretization as a mechanism of change in psychodrama and uses three terms that first require explanation. *Transportation* refers to relocating (or “re-presenting”) a realistic scene from a remembered “there-and-then” to the “here-and-now” in therapy. Transportation is contextualized by place, time, and characters present in the scene. *Transformation* refers to changing abstract and formless content into an externalized, tangible, embodied, and formed presentation. Transformation involves content that is not necessarily bound to a specific scene (e.g., place, time, characters), and hence is somewhat a-contextual. *Translation* refers to converting symbolic content into a realistic scene in the here-and-now. Thus, translation reflects the shift between transportation to transformation and vice-versa.

### The Pathway of Realistic Concretization

This pathway of concretization focuses on realistic conflicts from the protagonist’s daily life, which may be close to or distant from the protagonist’s core problem. This pathway consists of four phases. (1) Capturing the potentially emotionally loaded content from the brief interview that follows the warm-up phase and before moving to the action phase: the therapist focuses on the most important information from the protagonist’s brief interview (verbal and nonverbal information) when both the therapist and the protagonist try to clarify the protagonist’s content in a specific reality-based scene. Note that the brief interview may include deeper content related to the protagonist’s problem that the therapist will choose to address later in the session or the therapy process. (2) *Transportation* of realistic conflicts: the therapist and the protagonist will transport (i.e., relocate or “re-present”) the realistic scene, which contains the essential part of the protagonist’s problem, from a remembered “there-and-then” to the “here-and-now” in therapy. (3) Shared observation of the protagonist’s represented content: the therapist and the protagonist observe the realistic scene from the outside (from a distance) by using the “mirror” technique when the protagonist is played by a “double.” Landy (1983) considered that distancing can be physical, emotional, and cognitive; it can be from another person, a situation, or parts of one’s self. Distancing enables the protagonist to move from a participant to an observer role and thereby maintain an esthetic balance “between the two states of separation and closeness” (p. 175). (4) Emerged action insight is externalized, embodied, and integrated: because the protagonist moves back and forth from a participant to an observer role, witnesses and reevaluates the scene (Blatner, 2000), action insight emerges, i.e., experiential, action-generated, self-understanding, and awareness of the underlying sources of emotional, cognitive, or behavioral responses and difficulties in oneself or another person (Orkibi, 2020, personal communication). This action insight is internalized and reintegrated through the replay of the same scene or a new one. The essential component of reintegration is consistent with the psychodramatic notion that catharsis of integration must come after catharsis of abreaction (Moreno and Fox, 1978, p. 546). Specifically, catharsis of integration helps the protagonist to constructively re-own feelings that may have been repressed, re-integrate what has been split off, and reclaim the vitality and power associated with previously disowned aspects of the self (Blatner, 1996).

### Illustration of Realistic Concretization

To summarize and illustrate realistic concretization, we take the example of a protagonist named Adam who during the brief interview that followed the warm-up phase shared information about feeling insecure at his workplace. After focusing on the insecure theme, both the therapist and protagonist identified a specific and meaningful memory scene during which Adam’s colleague rudely silenced him in front of all the department employees during their weekly meeting. The therapist together with Adam *transported* this scene from the there-and-then to the here-and-now of therapy. Through the exploration and elaboration of content (by using the ability to move from role-playing inside the scene to observing it from the outside), Adam managed to see his automatic need to apologize to avoid conflict (action insight). Next, this action insight was grounded by examining Adam’s new potential and more adaptive response to his colleague. The action insight could also be integrated into a new scene where Adam confronts his parents during a family dinner when he felt he was silenced by them.

### The Pathway of Symbolic Concretization

This pathway of concretization focuses on symbolic content through four main phases: (1) Capturing potentially emotionally loaded content from the brief interview that follows the warm-up phase: the therapist focuses on the protagonist’s abstract and formless symbolic content that often express a rich inner experience through minimal words (Azoulay and Orkibi, 2015) and come from the core of the protagonist’s problem. (2) Transformation of abstract and formless content into external, tangible, embodied, formed representation: the therapist and the protagonist explore and elaborate the protagonist’s symbolic content by means of embodiment, namely, through physical and body-based exploration (Koch and Fuchs, 2011, p. 276) that is multilayered and is not necessarily confined to a specific time, place, or characters, unlike the transportation of realistic conflicts. In this process, the symbolic content is gradually *transformed* from a formless content that needs to be “decoded” by both the therapist and the client to a deeper and more elaborate description, which eventually will turn into an external, tangible, and embodied form. This process echoes Blatner (2000) claim that concretization in psychodrama helps protagonists convert their abstract statements and metaphors into specific, enacted actualities (Blatner, 2000, p. 238). (3) Shared observation of the protagonist’s represented content: both therapist and the protagonist observe the symbolic representation from a distance, as the protagonist moves back and forth from a participant to an observer role and thereby maintains an esthetic balance, similarly to the realistic pathway above. (4) Emerged action insight is externalized, embodied, and integrated: in the symbolic pathway, the action insight is physically embodied to integrate it into the dramatic surplus reality in the here-and-now without being translated into a realistic scene. Rather, it focuses on the integration of the new understandings (i.e., insight) in a symbolic way, as illustrated below. The concretization mechanism of change is a useful way to address symbolic and abstract content, which often represents the protagonist’s core problem (Blatner, 2000; Fonseca, 2004). The ability to create a representation of content and action insight through embodiment is one of the key benefits of the arts therapies in general and psychodrama in particular.

### Illustration of Symbolic Concretization

To summarize and illustrate the symbolic concretization pathway, we take the example of a protagonist named Benjamin who shared an image during the brief interview that followed the warm-up phase: “I feel like I’m about to fall off the edge of a cliff every time I imagine my ex-wife.” The therapist, together with Benjamin, explore, elaborate, and then transform the “cliff-edge” image into an external, tangible, and embodied presentation. This phase can contain verbal exploration by using leading questions about the feelings and thoughts the protagonist has at the edge of the cliff. They may also explore the protagonist’s bodily experience and what exists at the bottom of the cliff. This helps the protagonist gain action insights: Benjamin does not allow anyone to help him “get off the cliff” because he perceives help-seeking as an embarrassing weakness. Next, the protagonist’s action insight will be integrated into the same embodied symbolic scene, but a self-compassion voice will be introduced as a double by the therapists or auxiliary ago.

### The Pathway of Integrated Concretization

This pathway of concretization focuses on the protagonist’s gradual transition from content in the periphery – often emerging at the beginning of the therapy – toward content from the core of the protagonist’s problem that often unfolds during the therapy in a spiral. In contrast to the two pathways presented above, the integrated pathway of concretization addresses two types of content in a sequential process. Therapy can begin with the transportation of realistic conflicts that will later be *translated* into the symbolic scene and vice versa. The integrated pathway goes through five phases: (1) Capturing potentially emotionally loaded content from the interview. (2) *Transportation* of realistic conflicts OR *Transformation* of abstract and formless content into an external, tangible, and embodied representation. (3) Shared observation of the protagonist’s represented content. (4) *Translation* to a symbolic scene OR *Translation* to the realistic scene. (5) Emerged action insight is externalized, embodied, and integrated. Each integrated pathway starts from the earlier phases of realistic OR symbolic pathways. The choice of whether to go through the integrated pathway or to continue with the current process is made at the end of phase 2, according to the protagonist’s ability and needs. This pathway allows the protagonist to move between the different types and layers of content to gradually approach the core of his or her problem. The core notion guiding this pathway is that the phases of concretization help bypass the protagonist’s defense mechanisms and increase the protagonist’s accessibility to realistic and symbolic content. The integrated pathway is likely to be implemented in lengthier psychodrama group sessions. Fonseca (2004) noted that a client’s self-observation of repressed parts is the result of a joint effort by the therapist and the client to concretize the same internal material. This joint effort includes the analysis of the client’s body language and verbal messages, which together reveal conscious and unconscious material (Fonseca, 2004). Thus, through concretization, a dramatic dimension can be created in which different layers of contents emerge (Moreno and Fox, 1987; Fonseca, 2004, p. 9). This pathway helps account for the interactions between the phases and their different effects, produced both individually and as part of the integrated process (Blatner, 2000, pp. 238–239).

### Illustration of Integrated Concretization

To summarize and illustrate the pathway of integrated concretization, we take the example of a protagonist named Maya who during the brief psychodrama interview after the warm-up phase shared details about her problematic relationship with her older brother. The session beings with a realistic concretization where Maya’s argument with her older brother is transported to the here-and-now of therapy. After role-playing inside the scene, as well as observing the scene from the outside, Maya realizes (i.e., action insight) that she feels “like a weak dwarf whose brother is a bigger and smarter giant.” This symbolic statement helps the therapist and Maya to translate the realistic content into a symbolic form and hence getting closer to the core of Maya’s problem (through transforming this symbolic content into an external, tangible, and embodied representation). However, this sequence could have been reversed: Maya could have started by sharing her feelings through a metaphor. In this case, the reality-based relationship with Maya’s older brother would only have emerged later. In both scenarios, action insight can emerge from representing the content. In sum, the integrated pathway allows for flexibility in the implementation of concretization while adjusting the concretization phase to the protagonist’s content and needs in the therapy.

### The Therapeutic Benefits of Concretization

Identifying the main gains derived from the use of concretization as a mechanism of change in psychodrama helps to break down the mechanism process into individual change factors that affect the protagonist. These gains can often be expressed immediately as the therapy session progresses at the end of a single therapy session and/or at the end of therapy, where they reflect the accumulative effect. To summarize, this study defined four main therapeutic gains deriving from the use of concretization as a mechanism of change. (1) Reducing the ambiguity of the problem: The physical enactment of the problem may provide the protagonist with initial relief, since it presents an opportunity to grasp the problem by observing, identifying, naming, and defining it. In this sense, concretization in psychodrama is somewhat akin to the photographic process where, in both cases, a single moment or single content is captured, which is part of a wider story that becomes more accessible and observable. Vander May (1981) noted that the externalizing of the protagonist’s content in itself promotes therapy (Vander May, 1981, p. 35). (2) Externalizing the protagonist’s problem: concretization as a mechanism of change serves to detach the protagonist from his/her problem. Azoulay and Orkibi (2015) viewed externalization in psychodrama as a therapeutic procedure that can provide important information about the client that cannot be expressed in words. Specifically, “externalization helps clients to objectify the problem and to dis-identify with it. As a result, they can perceive problems as changeable products of circumstances or interpersonal processes, rather than as caused by their fixed psychology or personality” (Azoulay and Orkibi, 2015, p. 11). (3) Enhancing the therapist-protagonist therapeutic bond: concretization enables both therapist and protagonist to stand together inside the protagonist’s dramatic scene. This shared position allows them to jointly observe the externalizing of the protagonist’s inner content and, in a way, to experience the dramatic occurrence together for the first time. The therapist is actively involved in externalizing the protagonist’s content, thus allowing him or her to be physically close to the occurrence while observing the scene alongside the protagonist; in fact, often, the therapist will try to be in the same body position as the protagonist. The therapist–client bond is the foundation of the therapeutic alliance and is highly significant for the therapeutic process and to achieving therapeutic goals (Orkibi et al., 2017; Adoni-Kroyanker et al., 2020). Kellermann (1992) underscored the positive influence of psychodrama techniques on the protagonist’s empowerment and cognitive aspects of the therapist–protagonist therapeutic alliance. (4) Bypassing the protagonist’s defense mechanisms: concretization as a mechanism of change may allow the protagonist to access repressed content and can make the therapy more open and easier. The protagonist’s exposure to new content can take place when the protagonist acts concretely and physically in psychodrama. This physical action helps neutralize negative thoughts that may be part of the protagonist’s defense mechanisms. Concretization is often useful in situations where the client tends to intellectualize (Vander May, 1981) and the therapist’s physical presence close to the protagonist may help deal with the protagonist’s resistance by providing support and a sense of comfort and security (Leveton, 2001). Unlike observation of Kellermann (1996), our findings do not suggest that concretization has negative effects on clients who suffer from somatic disorders likely because the interviewees pay close attention to the changing needs and abilities of the protagonist or they may be less experienced working with concretization on somatic clients. The therapeutic gains described by the therapists interviewed in this study may shed light on the reasons why concretization as a mechanism of change is effective and the specific contribution of each phase of the three concretization pathways.

### Limitations and Future Directions

First, the study consists of seven interviews conducted only in Israel. Furthermore, we relied on interviews with therapists, without observing groups or individual sessions. In addition, the material collected did not include interviews with the psychodrama protagonists or group members. These shortcomings could be remedied by conducting a larger study with different types of data sources and respondents. Whereas this study focused on concretization as a mechanism of change in the protagonist, future studies could investigate how concretization can induce change in other members of the psychodrama group.

Future research on concretization as a mechanism of change should consider conducting an empirical study to examine the therapeutic effect of concretization as a mechanism of change and the effect of each pathway separately. The effectiveness of concretization as a mechanism of change should also be examined in short-term therapies and crisis interventions. Finally, research that focuses on the characteristics of concretization should be conducted to compare the situation where the group members create a physical representation to concretization that uses objects and images to create a physical representation. This would help determine the therapeutic advantages of each. We believe that further research into the underlying mechanisms of change in psychodrama will contribute to further develop psychodrama both theoretically and clinically.

## Data Availability Statement

The original contributions presented in the study are included in the article/supplementary material, further inquiries can be directed to the corresponding author.

## Ethics Statement

The studies involving human participants were reviewed and approved by the Ethics Committee for Human Research, Faculty of Social Welfare and Health Sciences, University of Haifa. The patients/participants provided their written informed consent to participate in this study.

## Author Contributions

AK contributed to the study design, literature review, data collection and analysis, reporting and discussion. HO contributed to the study design, literature review, reporting, discussion, and supervision. Both the authors contributed to the article and approved the submitted version.

### Conflict of Interest

The authors declare that the research was conducted in the absence of any commercial or financial relationships that could be construed as a potential conflict of interest.

## Footnotes

^1^“Somatoform” is a psychological disorder that manifests as physical symptoms (psychosomatic).
